# Vitreomacular traction after phakic, pseudophakic, and triple DMEK surgery

**DOI:** 10.1007/s00417-022-05821-4

**Published:** 2022-09-15

**Authors:** Sarah B. Zwingelberg, Stephanie Bresgen, Claus Cursiefen, Friederike Schaub

**Affiliations:** 1 Department of Ophthalmology, Faculty of Medicine and University, Hospital of Cologne, University of Cologne, Kerpener Straße 62, 50937 Cologne, Germany; 2 University of Rostock, Department of Ophthalmology, Doberaner Str. 140, 18057 Rostock, Germany

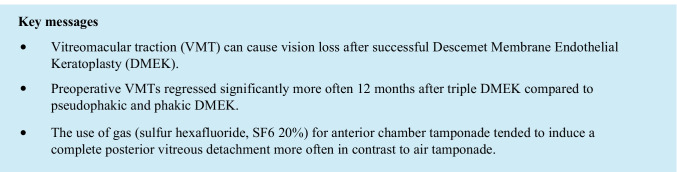



Dear Editor,

Posterior vitreous detachment (PVD) is a common phenomenon in the aging eye. In some cases (PVD) is incomplete and anomalous and may cause vitreomacular traction (VMT) with vision loss [1]. Known risk factors for developing vitreomacular traction are increasing age, female gender, the degree of myopia, and previous surgery. There are several treatment options: observation, pharmacologic vitreolysis, vitrectomy, or pneumatic vitreolysis [2].

Descemet membrane endothelial keratoplasty (DMEK) currently is the surgical treatment of choice in corneal endothelial diseases [3]. In DMEK, the anterior chamber is tamponaded with air or gas. To date, the incidence of VMT and the clinical course after corneal endothelial transplantation is unknown.

The purpose of this retrospective study was to analyze the incidence of anomalous PVD and the progression and development of VMTs after different types of DMEK surgery.

All consecutive eyes that underwent a first DMEK surgery between July 2011 and December 2018 at the Dept. of Ophthalmology at the University of Cologne with a preoperative macular examination by SD-OCT available and having a minimum follow-up of 1 year were included. All eyes underwent macular examination using SD-OCT (SPECTRALIS® HRA + OCT, Heidelberg Engineering GmbH, Heidelberg, Germany) preoperatively and as well as at each postoperative visit (1, 3, 6, 12 months). The specific scan protocol was a custom raster scan pattern with 37 Sects. (512 A-scans each) in a 30° × 20° field of view. Occurrence of VMT was defined as an incomplete, but partial PVD temporal to the fovea with traction on the fovea with edematous or cystic changes. For statistical analysis, Student’s *t*-test was performed.

A total of 1076 patients were eligible for the study, of which 57 eyes underwent a phakic DMEK (5.3%), 480 a pseudophakic DMEK (44.6%), and 539 a triple DMEK procedure The mean patient age overall cohort was 65.9 ± 6.3 years (50.1%).

The median follow-up time was 18.3 + 12.2 months. At the follow-up visit after 12 months in 62.9% OCT scans were available for evaluation.

The mean age of the total cohort was 65.9 ± 6.3 years. In the group of triple DMEK, mean age was 63.8 ± 8.5 years, in phakic DMEK 61.9 ± 5.9, and in pseudophakic DMEK 67.7 ± 7.7 years.

**Fig. 1:**
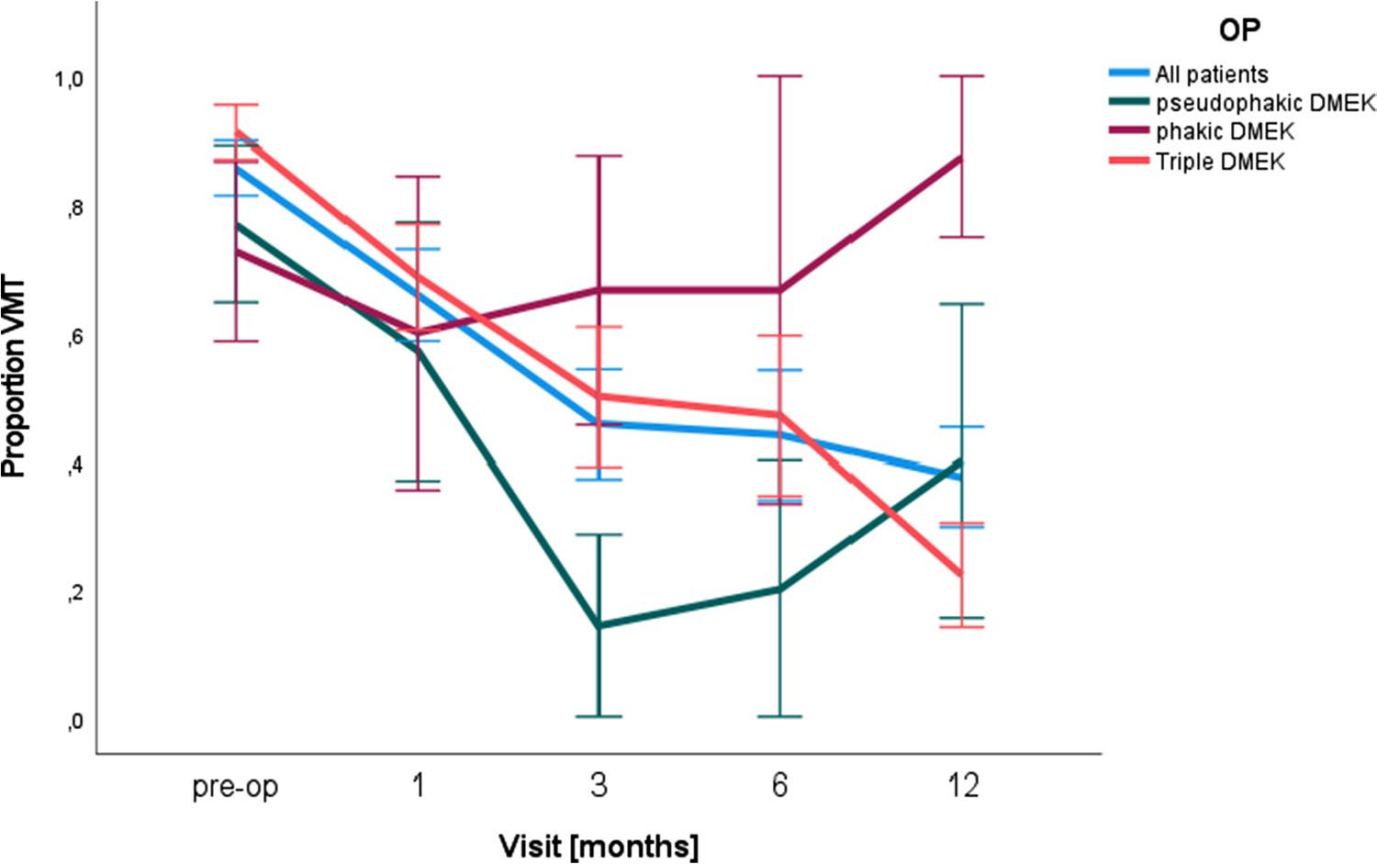
Triple DMEK leads to fastest resolution of VMT after endothelial keratoplasty. Proportion of VMT over the entire period of 12 months with a continuous decrease in all patients (blue curve). Patients with triple DMEK procedure (red curve) showed a significant and continuous decrease of VMTs after surgery

Preoperatively, 6.5% (*n* = 70) showed a VMT. Triple DMEK procedures (*n* = 46; 8.5%) showed a slightly increased preoperative incidence of VMT compared to pseudophakic (*n* = 13; 2.7%; *p* = 0.174) eyes. In the small sample of phakic DMEK (*n* = 11; 19.3%; *p* = 0.125), VMT was even higher. After 3 months, just 55% of the preoperative existing VMTs in triple DMEK were still detectable and reduced again after 6 months to 50% and finally decreased significantly to 22% after 12 months after DMEK surgery. In pseudophakic DMEK, the effect was also visible, especially postoperative: After 3 months, at least only 18% had a preoperative existing VMT with a decrease of the preoperative existing VMTs, but increased again 6 months after DMEK from 18 to 20% and after 12 months up to 35%, while also new VMTs developed during the postoperative course. In phakic DMEK, no significant change in the incidence of VMTs could be observed at any time. Especially in months 6 to 12, the VMTs showed an increase of 17%. Preoperative VMTs regressed in consequence significantly more often 12 months after triple DMEK compared to the other two types of surgery (*p* = 0.013). In contrast, 12 months after pseudophakic and phakic DMEK, there was a slightly increased incidence of VMT compared to preoperative incidence (pseudophakic: *p* = 0.091; phakic = 0.49, compare Fig. 1).

**Fig. 2:**
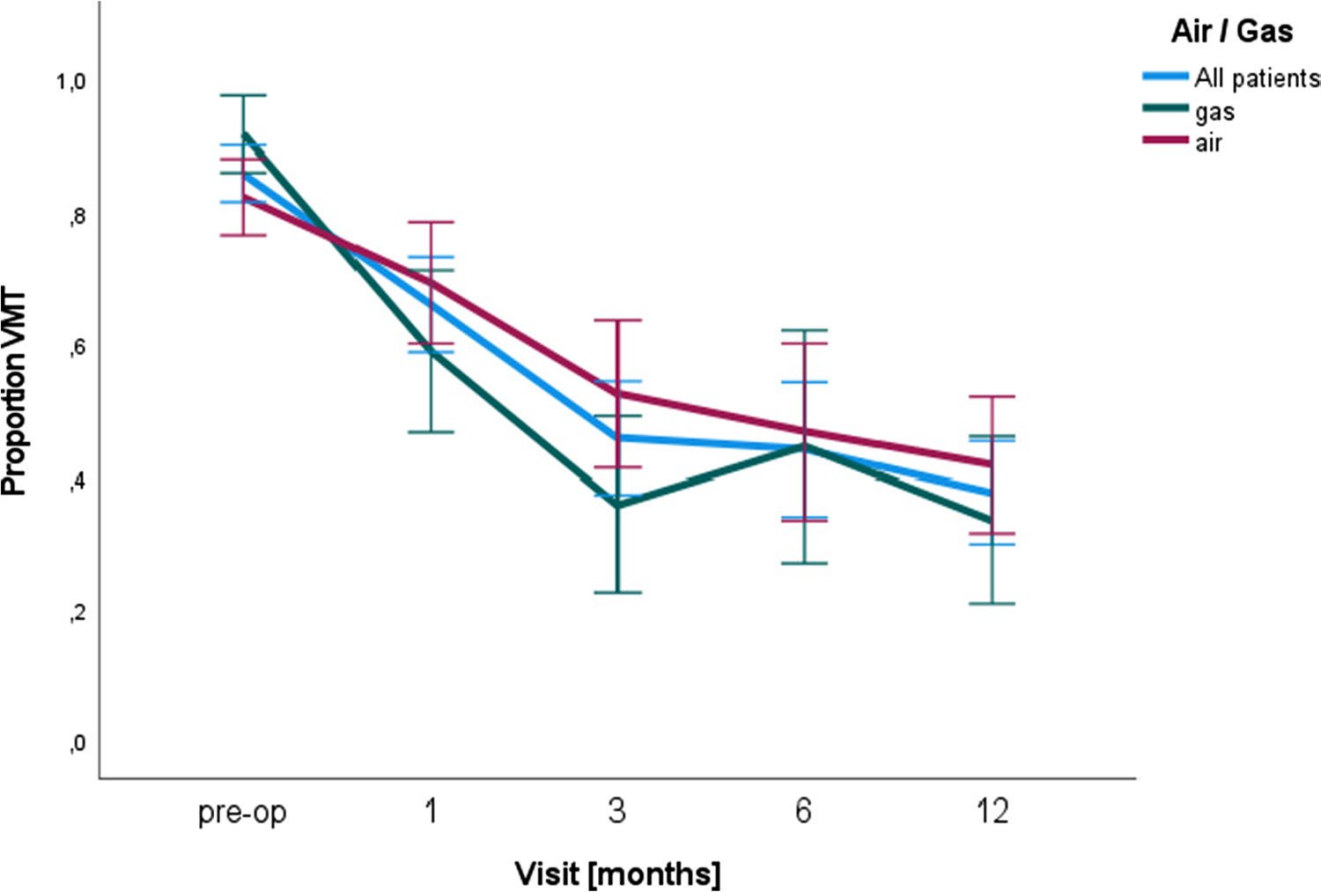
SF620% anterior chamber gas tamponade seems to support VMT resolution after DMEK. Proportion of VMT over the entire period of 12 months with a more significant decrease and successful PVD in eyes with gas (SF_6_ 20%) anterior chamber tamponade (green curve) compared to patients with air for graft attachment

The use of gas (sulfur hexafluoride, SF6 20%) for anterior chamber tamponade tended to induce a complete PVD more often, whereas following air tamponade, the incidence of VMT seemed to increase (compare Fig. 2).

All PVDs in eyes with VMT were uncomplicated during the course; in two cases, macular holes occurred within 3 months after a triple DMEK procedure by using air for anterior chamber tamponade.

According to the general decrease of VMT incidence after surgery shown in Fig. 1, it can be assumed that surgical manipulation in the anterior segment may favor a PVD and thereby VMT resolution. This observation has already been demonstrated in the procedure of cataract surgeries [4]. This fact could be the reason why the incidence of VMTs before and after surgery is higher in patients with phakic DMEK than in patients with a pseudophakic and triple DMEK. In addition, the intraocular temperature may also play a significant role. PVD can also be induced by thermal effects that may occur during phacoemulsification [5].

We could see a trend towards a higher resolving rate after anterior chamber gas tamponade. A potential explanation could be the more expansive properties leading to a higher rate of mechanical vitreous detachment [3].

Our study results represent the first data in a large cohort of eyes with corneal endothelial diseases including observations of the vitreomacular interface by OCT. The choice of surgery technique seems to have an impact on the resolution and the development of VMT, so that this could be taken into account during surgery planning. A more detailed analysis of the risk factors and clinical course of further vitreoretinal interface changes is desirable and should be evaluated in further studies.
